# *N*-Phenyl­pyrrolidine-1-carbothio­amide

**DOI:** 10.1107/S1600536808040907

**Published:** 2008-12-10

**Authors:** Jin-He Jiang

**Affiliations:** a Department of Chemistry and Chemical Engineering, Weifang University, Weifang 261061, People’s Republic of China

## Abstract

The title compound, C_11_H_14_N_2_S, was prepared by the reaction of 1-isothio­cyanato­benzene and pyrrolidine. In the crystal structure, inter­molecular N—H⋯S inter­actions are present.

## Related literature

For the applications of thio­amides, see: Toshiaki *et al.* (2003 ▶). For related structures, see: Casas *et al.* (2002 ▶); Cowley *et al.* (2002 ▶);
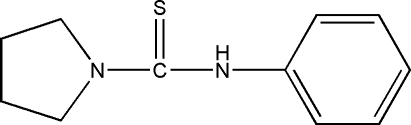


## Experimental

### 

#### Crystal data

C_11_H_14_N_2_S
*M* *_r_* = 206.30
Monoclinic, 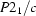

*a* = 11.195 (2) Å
*b* = 8.5694 (17) Å
*c* = 11.414 (2) Å
β = 108.03 (3)°
*V* = 1041.2 (4) Å^3^
*Z* = 4
Mo *K*α radiation
μ = 0.27 mm^−1^
*T* = 293 (2) K
0.25 × 0.20 × 0.18 mm

#### Data collection

Enraf–Nonius CAD-4 diffractometer
Absorption correction: none
4554 measured reflections
2393 independent reflections
2214 reflections with *I* > 2σ(*I*)
*R*_int_ = 0.018
3 standard reflections every 100 reflections intensity decay: none

#### Refinement

*R*[*F* ^2^ > 2σ(*F* ^2^)] = 0.045
*wR*(*F* ^2^) = 0.119
*S* = 1.30
2393 reflections
127 parameters
H-atom parameters constrained
Δρ_max_ = 0.30 e Å^−3^
Δρ_min_ = −0.46 e Å^−3^

### 

Data collection: *CAD-4 Software* (Enraf–Nonius, 1989 ▶); cell refinement: *CAD-4 Software*; data reduction: *NRCVAX* (Gabe *et al.*, 1989 ▶); program(s) used to solve structure: *SHELXS97* (Sheldrick, 2008 ▶); program(s) used to refine structure: *SHELXL97* (Sheldrick, 2008 ▶); molecular graphics: *SHELXTL* (Sheldrick, 2008 ▶); software used to prepare material for publication: *WinGX* (Farrugia, 1999 ▶).

## Supplementary Material

Crystal structure: contains datablocks global, I. DOI: 10.1107/S1600536808040907/at2689sup1.cif

Structure factors: contains datablocks I. DOI: 10.1107/S1600536808040907/at2689Isup2.hkl

Additional supplementary materials:  crystallographic information; 3D view; checkCIF report

## Figures and Tables

**Table 1:** Hydrogen-bond geometry (Å, °)

*D*—H⋯*A*	*D*—H	H⋯*A*	*D*⋯*A*	*D*—H⋯*A*
N1—H1*A*⋯S1^i^	0.86	2.64	3.4359 (17)	155

Symmetry code: (i) 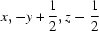.

**Fig. 1.**
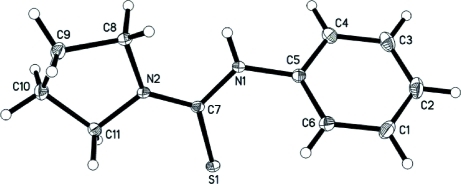
The structure of the title compound showing 30% probability displacement ellipsoids and the atom-numbering scheme.

## supplementary crystallographic information

### Comment

Thioamides have received considerable attention in the literature. They are attractive from several points of view in application (Toshiaki *et al.*, 2003). As part of our search for new thioamide compounds we synthesized the title compound (I), and describe its structure here.

In (I) (Fig. 1), the C6—S1 bond length of 1.689 (2)Å is comparable with C—S bond [1.688 (2) Å] reported (Cowley *et al.*, 2002). The distance of N1—C6 [1.332 (2) Å] is similar to the distance of reported [1.349 (1) Å] (Casas *et al.*, 2002). The crystal strucure is stabilized by intermolecular C—H···S interactions.

### Experimental

A mixture of the 1-isothiocyanatobenzene (0.1 mol), and pyrrolidine (0.1 mol) was stirred in refluxing ethanol (20 mL) for 4 h to afford the title compound (0.080 mol, yield 80%). Single crystals suitable for X-ray measurements were obtained by recrystallization from ethanol at room temperature.

### Refinement

H atoms were fixed geometrically and allowed to ride on their attached atoms, with C—H = 0.93 and 0.97 Å and N—H = 0.86 Å, and with *U*_iso_=1.2U_eq_(C,N).

### Crystal data

**Table:**

C_11_H_14_N_2_S	*F*(000) = 440
*M**_r_* = 206.30	*D*_x_ = 1.316 Mg m^−^^3^
Monoclinic, *P*2_1_/*c*	Mo *K*α radiation, λ = 0.71073 Å
Hall symbol: -P 2ybc	Cell parameters from 25 reflections
*a* = 11.195 (2) Å	θ = 1.8–27.0°
*b* = 8.5694 (17) Å	µ = 0.27 mm^−^^1^
*c* = 11.414 (2) Å	*T* = 293 K
β = 108.03 (3)°	Block, colourless
*V* = 1041.2 (4) Å^3^	0.25 × 0.20 × 0.18 mm
*Z* = 4	

### Data collection

**Table:**

Enraf–Nonius CAD-4 diffractometer	*R*_int_ = 0.018
Radiation source: fine-focus sealed tube	θ_max_ = 27.5°, θ_min_ = 1.9°
graphite	*h* = −14→14
ω scans	*k* = −11→11
4554 measured reflections	*l* = −14→14
2393 independent reflections	3 standard reflections every 100 reflections
2214 reflections with *I* > 2σ(*I*)	intensity decay: none

### Refinement

**Table:**

Refinement on *F*^2^	Primary atom site location: structure-invariant direct methods
Least-squares matrix: full	Secondary atom site location: difference Fourier map
*R*[*F*^2^ > 2σ(*F*^2^)] = 0.045	Hydrogen site location: inferred from neighbouring sites
*wR*(*F*^2^) = 0.119	H-atom parameters constrained
*S* = 1.30	*w* = 1/[σ^2^(*F*_o_^2^) + (0.0574*P*)^2^ + 0.3194*P*] where *P* = (*F*_o_^2^ + 2*F*_c_^2^)/3
2393 reflections	(Δ/σ)_max_ < 0.001
127 parameters	Δρ_max_ = 0.30 e Å^−^^3^
0 restraints	Δρ_min_ = −0.46 e Å^−^^3^

### Special details

**Table:**

Geometry. All e.s.d.'s (except the e.s.d. in the dihedral angle between two l.s. planes) are estimated using the full covariance matrix. The cell e.s.d.'s are taken into account individually in the estimation of e.s.d.'s in distances, angles and torsion angles; correlations between e.s.d.'s in cell parameters are only used when they are defined by crystal symmetry. An approximate (isotropic) treatment of cell e.s.d.'s is used for estimating e.s.d.'s involving l.s. planes.
Refinement. Refinement of *F*^2^ against ALL reflections. The weighted *R*-factor *wR* and goodness of fit *S* are based on *F*^2^, conventional *R*-factors *R* are based on *F*, with *F* set to zero for negative *F*^2^. The threshold expression of *F*^2^ > σ(*F*^2^) is used only for calculating *R*-factors(gt) *etc*. and is not relevant to the choice of reflections for refinement. *R*-factors based on *F*^2^ are statistically about twice as large as those based on *F*, and *R*- factors based on ALL data will be even larger.

### Fractional atomic coordinates and isotropic or equivalent isotropic displacement parameters (Å^2^)

**Table:**

	*x*	*y*	*z*	*U*_iso_*/*U*_eq_	
S1	0.50030 (4)	0.12695 (5)	0.27663 (4)	0.01992 (15)	
N2	0.34546 (13)	0.29432 (17)	0.09833 (13)	0.0162 (3)	
N1	0.53176 (13)	0.23342 (19)	0.06683 (13)	0.0183 (3)	
H1A	0.4979	0.2678	−0.0068	0.022*	
C7	0.45718 (15)	0.2256 (2)	0.14190 (15)	0.0152 (3)	
C5	0.66016 (16)	0.1894 (2)	0.10058 (16)	0.0177 (4)	
C8	0.30485 (17)	0.3898 (2)	−0.01429 (16)	0.0200 (4)	
H8A	0.2822	0.3248	−0.0874	0.024*	
H8B	0.3704	0.4617	−0.0182	0.024*	
C11	0.25255 (16)	0.2964 (2)	0.16519 (16)	0.0203 (4)	
H11A	0.2806	0.3614	0.2382	0.024*	
H11B	0.2364	0.1919	0.1892	0.024*	
C4	0.70097 (18)	0.0997 (2)	0.01900 (18)	0.0225 (4)	
H4A	0.6439	0.0646	−0.0541	0.027*	
C9	0.19114 (17)	0.4771 (2)	−0.00189 (18)	0.0245 (4)	
H9A	0.1312	0.4984	−0.0821	0.029*	
H9B	0.2158	0.5748	0.0418	0.029*	
C6	0.74613 (17)	0.2436 (2)	0.20885 (17)	0.0241 (4)	
H6A	0.7196	0.3065	0.2624	0.029*	
C2	0.91276 (19)	0.1125 (3)	0.1562 (2)	0.0337 (5)	
H2A	0.9971	0.0854	0.1755	0.040*	
C3	0.82751 (19)	0.0628 (2)	0.0475 (2)	0.0307 (5)	
H3A	0.8551	0.0038	−0.0075	0.037*	
C10	0.13624 (17)	0.3649 (2)	0.07178 (18)	0.0243 (4)	
H10A	0.0844	0.4197	0.1124	0.029*	
H10B	0.0865	0.2841	0.0193	0.029*	
C1	0.87165 (18)	0.2032 (3)	0.23648 (19)	0.0309 (5)	
H1B	0.9289	0.2375	0.3098	0.037*	

### Atomic displacement parameters (Å^2^)

**Table:**

	*U*^11^	*U*^22^	*U*^33^	*U*^12^	*U*^13^	*U*^23^
S1	0.0211 (2)	0.0247 (3)	0.0136 (2)	0.00322 (17)	0.00489 (16)	0.00532 (16)
N2	0.0159 (7)	0.0202 (7)	0.0125 (6)	0.0012 (6)	0.0045 (5)	0.0028 (5)
N1	0.0162 (7)	0.0264 (8)	0.0124 (6)	0.0025 (6)	0.0049 (5)	0.0027 (6)
C7	0.0164 (8)	0.0160 (8)	0.0127 (7)	−0.0019 (6)	0.0036 (6)	−0.0021 (6)
C5	0.0180 (8)	0.0180 (8)	0.0175 (8)	0.0006 (7)	0.0060 (7)	0.0036 (6)
C8	0.0204 (8)	0.0248 (9)	0.0150 (8)	0.0033 (7)	0.0056 (6)	0.0049 (7)
C11	0.0174 (8)	0.0270 (10)	0.0181 (8)	−0.0008 (7)	0.0078 (7)	0.0009 (7)
C4	0.0254 (9)	0.0195 (9)	0.0248 (9)	0.0001 (7)	0.0108 (7)	−0.0009 (7)
C9	0.0204 (9)	0.0264 (10)	0.0264 (9)	0.0051 (7)	0.0070 (7)	0.0076 (8)
C6	0.0223 (9)	0.0304 (10)	0.0192 (9)	−0.0014 (8)	0.0057 (7)	0.0016 (7)
C2	0.0200 (9)	0.0385 (12)	0.0436 (12)	0.0075 (8)	0.0111 (9)	0.0164 (10)
C3	0.0298 (10)	0.0266 (10)	0.0423 (12)	0.0080 (8)	0.0206 (9)	0.0029 (9)
C10	0.0176 (8)	0.0299 (10)	0.0262 (9)	0.0017 (7)	0.0078 (7)	0.0028 (8)
C1	0.0201 (9)	0.0415 (12)	0.0264 (10)	−0.0039 (8)	0.0002 (8)	0.0098 (9)

### Geometric parameters (Å, °)

**Table:**

S1—C7	1.6892 (17)	C4—C3	1.388 (3)
N2—C7	1.332 (2)	C4—H4A	0.9300
N2—C11	1.468 (2)	C9—C10	1.527 (3)
N2—C8	1.472 (2)	C9—H9A	0.9700
N1—C7	1.371 (2)	C9—H9B	0.9700
N1—C5	1.419 (2)	C6—C1	1.385 (3)
N1—H1A	0.8600	C6—H6A	0.9300
C5—C4	1.389 (2)	C2—C3	1.379 (3)
C5—C6	1.390 (3)	C2—C1	1.384 (3)
C8—C9	1.520 (2)	C2—H2A	0.9300
C8—H8A	0.9700	C3—H3A	0.9300
C8—H8B	0.9700	C10—H10A	0.9700
C11—C10	1.522 (3)	C10—H10B	0.9700
C11—H11A	0.9700	C1—H1B	0.9300
C11—H11B	0.9700		
			
C7—N2—C11	123.08 (14)	C5—C4—H4A	120.2
C7—N2—C8	124.92 (14)	C8—C9—C10	103.46 (15)
C11—N2—C8	111.72 (13)	C8—C9—H9A	111.1
C7—N1—C5	125.40 (15)	C10—C9—H9A	111.1
C7—N1—H1A	117.3	C8—C9—H9B	111.1
C5—N1—H1A	117.3	C10—C9—H9B	111.1
N2—C7—N1	115.37 (15)	H9A—C9—H9B	109.0
N2—C7—S1	122.14 (13)	C1—C6—C5	119.56 (19)
N1—C7—S1	122.40 (13)	C1—C6—H6A	120.2
C4—C5—C6	119.98 (17)	C5—C6—H6A	120.2
C4—C5—N1	118.73 (16)	C3—C2—C1	119.36 (19)
C6—C5—N1	121.13 (16)	C3—C2—H2A	120.3
N2—C8—C9	103.50 (14)	C1—C2—H2A	120.3
N2—C8—H8A	111.1	C2—C3—C4	120.80 (19)
C9—C8—H8A	111.1	C2—C3—H3A	119.6
N2—C8—H8B	111.1	C4—C3—H3A	119.6
C9—C8—H8B	111.1	C11—C10—C9	103.05 (15)
H8A—C8—H8B	109.0	C11—C10—H10A	111.2
N2—C11—C10	103.34 (14)	C9—C10—H10A	111.2
N2—C11—H11A	111.1	C11—C10—H10B	111.2
C10—C11—H11A	111.1	C9—C10—H10B	111.2
N2—C11—H11B	111.1	H10A—C10—H10B	109.1
C10—C11—H11B	111.1	C2—C1—C6	120.7 (2)
H11A—C11—H11B	109.1	C2—C1—H1B	119.6
C3—C4—C5	119.51 (18)	C6—C1—H1B	119.6
C3—C4—H4A	120.2		

### Hydrogen-bond geometry (Å, °)

**Table:**

*D*—H···*A*	*D*—H	H···*A*	*D*···*A*	*D*—H···*A*
N1—H1A···S1^i^	0.86	2.64	3.4359 (17)	155

Symmetry codes: (i) *x*, −*y*+1/2, *z*−1/2.
